# Maternal Pregnancy Intention and Antenatal Care Seeking Behaviors in Bangladesh: Evidence From Bangladesh Demographic and Health Survey, 2018

**DOI:** 10.3389/ijph.2023.1605944

**Published:** 2023-07-11

**Authors:** Md. Hafizul Islam, Ahmed Jubayer, Md. Moniruzzaman Nayan, Abira Nowar, Saiful Islam

**Affiliations:** ^1^ Institute of Nutrition and Food Science, University of Dhaka, Dhaka, Bangladesh; ^2^ Bangladesh Institute of Social Research (BISR) Trust, Dhaka, Bangladesh

**Keywords:** pregnant women, pregnancy intention, unintended pregnancy, antenatal care, Bangladesh

## Abstract

**Objective:** This study aimed to examine the association between pregnancy intention and antenatal care (ANC)-seeking behaviors among women in Bangladesh.

**Methods:** ANC-related data of 5,012 women, from the 2018 Bangladesh Demographic and Health Survey (BDHS), who had a live birth within 3 years preceding the survey were analyzed in the study. Multivariate logistic regression models were used to assess the association of pregnancy intention with ANC utilization.

**Results:** Approximately one-fifth (20.9%) of the women had unintended pregnancy. Among all the women, 40.4% received their first ANC visit within the first three months of pregnancies, 47% had at least four ANC visits, 26.1% received all the components of ANC services, and 22.2% received an adequate dosage of supplementary iron-folic acid tablets/syrup. Women with unintended pregnancy were less likely to receive their first ANC visit within the first 3 months, four or more ANC visits, and all ANC services than those with intended pregnancy.

**Conclusion:** Unintended pregnancy was inversely associated with the proper utilization of ANC among women in Bangladesh. Appropriate measures to reduce unintended pregnancy might foster the utilization of optimum antenatal care.

## Introduction

All the member countries of the United Nations (UN) including Bangladesh pledged to reduce the maternal mortality rate to less than 70 deaths per 100,000 live births by 2030 [1]. However, pregnancy-related preventable morbidity and mortality still remain unacceptable in many low- and middle-income countries (LMICs) like Bangladesh [2]. It is evident that this high maternal morbidity and mortality rate is associated with inadequate maternal pregnancy care [2]. Moreover, maternal mortality, pregnancy-related complications, and adverse pregnancy outcomes could substantially be averted through early detection of complications and ensuring proper pregnancy care [3–5].

The World Health Organization (WHO) envisions a world where “every pregnant woman and newborn receives quality care throughout the pregnancy, childbirth, and the postnatal period” [6]. WHO recommends that adequate healthcare for a normal pregnancy that has no complications should have at least four Antenatal Care (ANC) visits from a skilled healthcare professional, with the first visit occurring within the first trimester (first 3 months of pregnancy) [7]. ANC comprises the care provided by a qualified doctor, nurse, midwife, family welfare visitor, community skilled birth attendant, medical assistant, etc. to pregnant women to ensure the best health conditions for both mother and baby during pregnancy [5]. Moreover, during these visits, the mothers should have several health services, including taking body weight, checking blood pressure, testing blood samples, testing urine samples, performing ultrasonograms, and giving information on pregnancy complications to the mother. In addition, women should take adequate dosage of iron/folic acid (IFA) supplementation during pregnancy, as inadequate intake of these nutrients may have a negative impact on maternal health, fetal development, and pregnancy outcomes [8–10].

In developing countries like Bangladesh, various factors including age, lower educational level of women and their partner [11–13], lower socio-economic status [11–14], higher parity [12–14], intimate partner violence [15], lack of women’s decision making power, [16] etc. contribute to lower use of antenatal care. Similarly, women with unintended pregnancies have been documented to make inadequate ANC visits and delay the initiation of the first visit [11, 14, 17, 18]. Pregnancies are considered unintended when the pregnancies are unwanted (i.e., when no children, or no more children, are desired) or mistimed (i.e., they occur earlier than expected). Besides, women with unintended pregnancies are supposed to have less attention from their partner, and family members which leads to lower use of self-care as well. Thus, poor utilization of ANC increases pregnancy related complications (i.e., unfavorable pregnancy outcome, maternal morbidity, and mortality) and obstetric complications (i.e., premature birth, low birth weight, and neonatal death) [3, 4].

Bangladesh aims to reduce the maternal mortality ratio to 121 deaths per 100,000 live births, and the neonatal mortality rate to 18 deaths per 1,000 live births by 2022 to achieve the goals of its government’s 4th Health, Population, and Nutrition Sector Program. The percentage of women receiving any ANC from medically trained persons has increased from 42% in 2004 to 82% in 2018, and at least four ANC visits have increased from 17% in 2004 to 47% in 2018 [19]. However, the use of modern contraceptives has been almost stagnant during the last decade (from 48% in 2004 to 52% in 2018), and unintended pregnancy is still prevailing here (21% in 2018) [19]. It is evident that women with unintended pregnancies are at greater risk of not taking adequate ANC visits and delaying the initiation of the first ANC visits in Bangladesh [11, 14, 20].

We found two studies [11, 14] that reported the effect of unintended pregnancy on the use of ANC among Bangladeshi women. However, they focused on a limited number of ANC components, namely, receiving at least one ANC visit, and the required number of ANC visits. These studies did not include the timely initiation of first ANC visit and the quality of ANC services which are recommended by the WHO. The studies also did not conduct any predictive analysis for taking supplementary IFA during pregnancy, a crucial component of ANC for preventing anemia during pregnancy. Therefore, a further comprehensive investigation is required for better insights covering all the important indicators of ANC in Bangladesh. To extend the previous work and fill this knowledge gap, we conducted the current study to examine the association between pregnancy intention and ANC-seeking behaviors (timing of the initiation of the first ANC visit, adequacy of ANC visits, and coverage of all ANC services) among women in Bangladesh using the latest nationally representative data. While defining the quality of ANC coverage, we included all six recommended items of ANC services. Additionally, the current study assessed the practice of taking an adequate dosage of IFA supplements and explored its association with pregnancy intention. The study also revealed the factors of unintended pregnancy in Bangladesh. Thus, the study findings might contribute to policy design to reduce unintended pregnancy and augment the uptake of proper ANC.

## Methods

### Data Source

This study used nationally representative cross-sectional data from the Bangladesh Demographic and Health Survey (BDHS) carried out in 2018. The selection of households and data collection were conducted based on a two-stage stratified sample of households. In the first stage of sampling, 675 enumeration areas (EA) (250 in urban areas and 425 in rural areas) were selected with probability proportional to EA size. In the second stage, a systematic sample of an average of 30 households per EA was selected to provide statistically reliable estimates of all eight divisions and rural and urban areas separately. Finally, the survey collected data from 672 clusters (EA) covering all eight divisions of Bangladesh. Further details of the survey were described previously [19].

### Sample Selection

**FIGURE 1:**
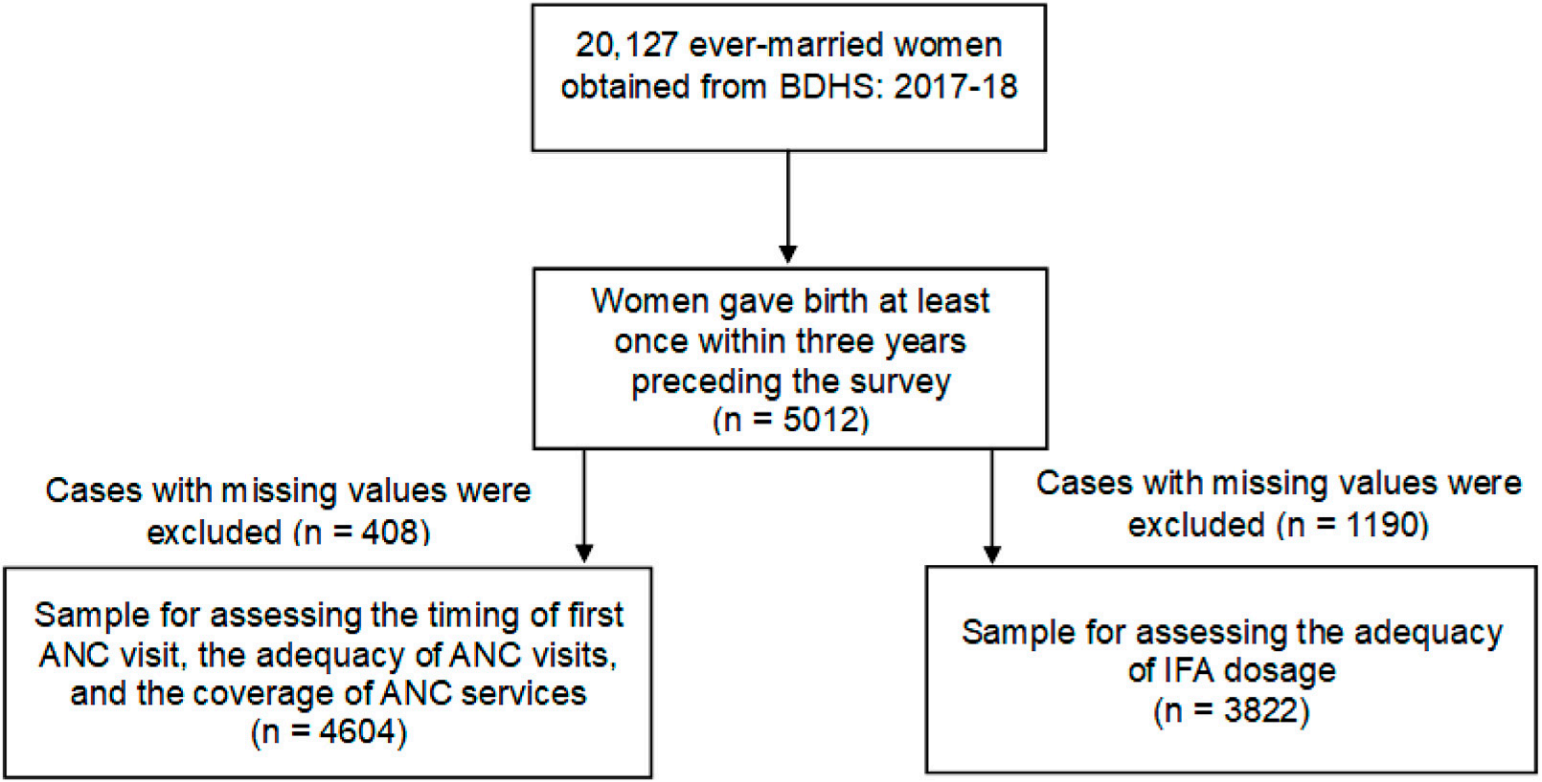
Flow chart showing the inclusion of the study participants (Bangladesh, 2018).

A total of 20,127 ever-married women (15–49 years) from 19,457 households were interviewed in this survey. The women who gave birth at least once within 3 years preceding the survey and responded to questions on pregnancy intention and antenatal care were considered for the analysis. Finally, data from 5,012 eligible women were analyzed in the present study (Figure 1).

### Outcome Variables

The outcome variables of the study were four indicators of ANC. These indicators were: 1) timing of the initiation of the first ANC visit, 2) adequacy of the ANC visits, 3) coverage of the ANC services, and 4) taking adequate dosage of IFA supplements. The first outcome variable was defined as whether the women received their first ANC visit/service within the first 3 months of their pregnancy from a qualified doctor, nurse, midwife, paramedic, family welfare visitor, community skilled birth attendant, medical assistant, or sub-assistant community medical officer [7]. The adequacy of ANC visits was considered when they received four or more ANC visits [7]. The coverage of ANC services was defined as receiving all six recommended items of ANC services during the ANC visits. These six items of ANC were taking body weight, checking blood pressure, testing blood and urine sample, performing an ultrasonogram, and giving information on pregnancy complications to the women. They were asked whether they received these services at least once during their pregnancy, but the frequency of receiving these services was not considered. Another outcome variable, taking adequate IFA supplements, was defined as whether they received adequate IFA tablets during their pregnancy. The recommended minimum dosage of IFA tablets is ≥180 (once daily after the first trimester) [21]. Consumption of 180 or more IFA tablets was defined as adequate and <180 as inadequate.

### Predictor Variables

The main predictive variable of this study was the “intention of the mothers towards their last pregnancies (intended/unintended).” This variable was measured in the 2018 BDHS by asking mothers to recall their feelings at the time of conception for each baby born within the past 3 years. Women were asked whether their births/pregnancies were wanted at the time (planned birth), at a later time (mistimed birth), or not at all (unwanted birth). This study used this predictive variable as a dichotomous one with two categories. Those who wanted to be pregnant at that time were categorized as “intended,” and those who wanted to be pregnant later or not at all were categorized as “unintended.”

### Control Variables

Several background characteristics at the individual and household levels were included in the analyses as potential confounders that have been identified from previous studies to be associated with receiving ANC in Bangladesh and other LMICs [11–14, 16, 20, 22, 23]. At the individual level, these variables were the age of the women (15–24 years, 25–34 years, and 35–49 years), educational level of the women (no formal education, primary, secondary, and higher), educational level of their partner/husband (no formal education, primary, secondary, and higher), employment status of the women, parity, and the use of contraception. Family size (≤5 and >5) and wealth quintiles (poorest, second, middle, fourth, and richest) were included as potential confounding variables at the household level. Moreover, the type of residence (rural or urban) and region (division) were also included.

### Statistical Analyses

Maternal pregnancy intention and other individual- and household-level background characteristics were summarized as frequencies and percentages. The 2018 BDHS used sampling weights for individual women, and in this study, all the analyses were adjusted for the sampling weights. A chi-square test was done to find the association between maternal pregnancy intention and other individual- and household-level background characteristics with ANC.

### Regression Model Building

The components of ANC were binary variables: timing of the initiation of the first ANC visit [before 3 months, after 3 months], adequacy of ANC visits [less than four visits, at least four visits], coverage of all the ANC services [yes, no], and taking adequate supplementary IFA tablets/syrup [yes, no]. Four different multivariate logistic regression models were used to find the relationship between maternal pregnancy intention and other socio-demographic variables with the four components of ANC. Another logistic regression model was used to find the potential socio-demographic factors of unintended pregnancy [yes, no]. To find the effect of socio-demographic variables in receiving adequate ANC separately among women with intended and unintended pregnancies, eight more logistic regression models were used. Variables with significant associations at *p*-values <0.25 in bivariate analyses were considered for inclusion in the regression models [24]. The underlying assumptions of the logistic regression models were examined before the final model building. The model’s multicollinearity was checked using the variance inflation factor (VIF), and a VIF value greater than 2 was regarded as evidence of multicollinearity [25]. *p*-values <0.05 were used to determine whether a variable was statistically significant. The stepwise forward entry method was used. The regression model’s fit was assessed using Pearson’s goodness of fit statistic. The association was reported as an adjusted odds ratio (AOR) with a 95% confidence interval (CI). IBM Statistical Package for Social Science (SPSS), version 25, was used to perform all statistical analyses.

## Results

### Socio-Demographic Characteristics of the Respondents

Among 5,012 women, 79.1% reported their last pregnancy as intended, while for 20.9% of women their last pregnancy was unintended (wanted later/wanted not at all) (Table 1). A higher proportion of women were between 15 and 24 years (53.1%), belonging to rural areas (73.2%), from Dhaka division (25.6%), and without any employment (62.7%). Most of the women and their partners had primary to secondary education (76.6% and 66.8%, respectively), while only 17%–18% had formal education of higher secondary or above. Approximately two-thirds of them (66.2%) used different methods of contraception. The mean number of children ever born was 2.1 (SD = 1.25), and nearly 70% had given birth to one or two children.

**TABLE 1:** Pregnancy intention and socio-demographic characteristics of the women aged 15–49 years who had a live birth in the 3 years preceding the survey (*n* = 5,012) (Bangladesh, 2018).

Variables	n (%)
Intention for pregnancy	
Intended	3,954 (79.1)
Unintended	1,058 (20.9)
Age of the women (in years)	
15–24	2,642 (53.1)
25–34	2,059 (41.0)
≥35	311 (5.9)
Area of residence	
Rural	3,287 (73.2)
Urban	1,725 (26.8)
Region (Division)	
Barisal	533 (5.7)
Chattagram	835 (21.2)
Dhaka	741 (25.6)
Khulna	542 (9.2)
Mymensingh	603 (8.5)
Rajshahi	527 (11.6)
Rangpur	559 (10.6)
Sylhet	690 (7.6)
Educational level of the women	
No formal education	312 (6.3)
Primary	1,392 (27.6)
Secondary	2,402 (49.0)
Higher secondary	906 (17.1)
Employment status of women	
Yes	1,880 (37.3)
No	3,132 (62.7)
Educational level of the partner	
No formal education	679 (13.5)
Primary	1,657 (33.2)
Secondary	1,635 (33.6)
Higher secondary	962 (18.2)
Don’t know/Missing	79 (1.6)
Wealth quintile	
Poorest	1,079 (20.6)
Second	1,017 (20.5)
Middle	905 (19.2)
Fourth	988 (20.2)
Richest	1,023 (19.5)
Parity	
1	1,915 (38.2)
2	1,638 (32.8)
3	855 (16.7)
≥4	604 (12.2)
Family size	
≤5	2,524 (51.1)
>5	2,488 (48.9)
Use of contraception	
Yes	3,360 (66.2)
No	1,652 (33.8)

### Socio-Demographic Factors of Unintended Pregnancy

Age of the women, residence area, educational level of the women, wealth index, family size, and current use of contraception were significantly associated with unintended pregnancy (Supplementary Table S1). Women aged 25–34 years (AOR: 1.39, 95% CI: 1.19–1.61, *p* < 0.001) and ≥35 years (AOR: 2.81, 95% CI: 2.15–3.77, *p* < 0.001) were more likely to have unintended pregnancy compared to women of 15–24 years. Unintended pregnancy was less likely among women from urban areas (AOR: 0.81, 95% CI: 0.68–0.95, *p* = 0.010) compared to their peers in rural areas. Higher educational levels and higher wealth index were associated with lower odds of unintended pregnancy among women. On the other hand, women having large family sizes (>5 members) had more chance (AOR: 1.46, 95% CI: 1.27–1.69, *p* < 0.001) to have an unintended pregnancy than women having small family sizes (≤5 members). Those currently using the contraception method had more chance (AOR: 1.52, 95% CI: 1.30–1.79, *p* < 0.001) to have their last pregnancy unintended.

### Use of Antenatal Care

**FIGURE 2:**
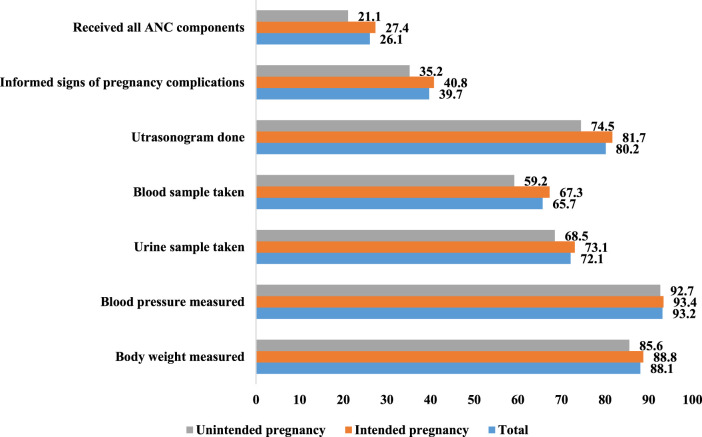
Uptake of antenatal care services (%) among the women aged 15–49 years who had a live birth in the 3 years preceding the survey (*n* = 4,604) (Bangladesh, 2018).

Approximately one in ten (8%) of the women (not shown) did not have any ANC visits during their pregnancy. Among those who had at least one ANC visit, 40.4% had their first visit within the first 3 months of their pregnancy (Table 2). Approximately half (47%) of them had at least four ANC visits, and more than three-fourths of them (76.4%) received IFA supplements at least once during the pregnancy period (not shown). However, the adequacy of receiving IFA supplement was among only about one-fifth of them (22.2%) (Table 2). Figure 2 shows the coverage of ANC services; body weight measured (88.1%), blood pressure measured (93.2%), urine sample taken (72.1%), blood sample taken (65.7%), ultrasonogram done (80.2%), and informed signs of pregnancy complications (39.7%). The percentage of women receiving ANC services was comparatively higher among women with their intended pregnancy compared to others: body weight measured (88.8% vs. 85.6%), blood pressure measured (93.4% vs. 92.7%), urine sample taken (73.1% vs. 68.5%), blood sample taken (67.1% vs. 59.2%), ultrasonogram done (81.7% vs. 74.5%), and informed signs of pregnancy complications (40.8% vs. 35.2%). Approximately one-fourth (26.1%) of them received all the components of ANC services, while this prevalence was 27.4% and 21.1% for women with intended and unintended pregnancies, respectively.

**TABLE 2:** Antenatal care and socio-demographic characteristics of the women aged 15–49 years who had a live birth in the 3 years preceding the survey (Bangladesh, 2018).

Variables	First ANC visit within 3 months n (%)	*p*-value*	Received at least 4 ANC visits n (%)	*p*-value*	Received all the ANC services n (%)	*p*-value*	Received adequate IFA n (%)	*p*-value*
Total	1915 (40.4)		2,414 (47.0)		1,238 (26.1)		830 (22.2)	
Intention for pregnancy								
Intended	1,587 (42.0)	<0.001	1,985 (49.2)	<0.001	1,036 (27.4)	<0.001	697 (23.3)	<0.001
Unintended	328 (33.9)		429 (38.7)		202 (21.1)		133 (17.8)	
Age of the women (in years)								
15–24	989 (39.9)		1,274 (47.1)		625 (25.2)		418 (21.1)	
25–34	819 (41.8)	0.13	1,010 (48.0)	0.05	547 (27.7)	0.031	359 (23.5)	0.137
≥35	107 (34.9)		130 (38.7)		66 (23.3)		53 (23.3)	
Area of residence							
Rural	1,099 (36.7)	<0.001	1,396 (42.7)	<0.001	661 (22.4)	<0.001	481 (20.1)	<0.001
Urban	816 (49.8)		1,018 (58.7)		577 (35.7)		349 (27.6)	
Region (division)								
Barisal	172 (35.9)		214 (37.9)		120 (25.7)		56 (16.2)
Chattagram	268 (34.6)		338 (38.6)		202 (25.1)		157 (25.1)	
Dhaka	353 (49.7)		395 (51.2)		228 (31.2)		158 (26.3)	
Khulna	212 (39.6)	<0.001	312 (57.0)	<0.001	154 (28.5)	0.001	93 (22.1)	<0.001
Mymensingh	226 (40.2)		291 (45.4)		130 (22.0)		116 (25.1)	
Rajshahi	185 (33.9)		266 (47.9)		121 (22.7)		70 (15.0)	
Rangpur	211 (35.9)		344 (59.2)		139 (23.6)		99 (19.5)	
Sylhet	288 (46.0)		254 (34.6)		144 (21.7)		81 (16.3)	
Educational level of the women								
No formal education	67 (27.0)		63 (19.6)		30 (13.3)		22 (12.9)	
Primary	371 (30.2)	<0.001	476 (34.1)	<0.001	212 (16.9)	<0.001	105 (11.7)	<0.001
Secondary	911 (39.3)		1,236 (50.1)		585 (25.4)		409 (22.3)	
Higher secondary	566 (61.6)		639 (69.1)		411 (44.7)		294 (37.2)	
Employment status of women							
Yes	620 (35.0)	<0.001	888 (45.5)	0.32	396 (22.2)	<0.001	271 (18.5)	0.001
No	1,295 (43.5)		1,526 (47.9)		842 (28.4)		559 (24.4)	
Educational level of the partner							
No formal education	156 (26.9)		203 (29.8)		89 (16.8)		62 (14.4)	
Primary	464 (31.1)	<0.001	637 (37.2)	<0.001	267 (17.4)	<0.001	181 (16.0)	<0.001
Secondary	669 (41.6)		849 (50.6)		445 (27.9)		279 (22.2)	
Higher secondary	599 (61.8)		694 (71.9)		437 (44.3)		301 (35.7)	
Wealth quintile								
Poorest	237 (26.9)		333 (30.9)		116 (13.0)		97 (15.1)	
Second	312 (33.5)		387 (36.4)		162 (16.6)		130 (17.8)	
Middle	328 (37.4)	<0.001	433 (45.5)	<0.001	207 (24.1)	<0.001	128 (18.3)	<0.001
Fourth	395 (40.1)		532 (51.8)		289 (30.6)		169 (21.8)	
Richest	643 (61.7)		729 (71.7)		464 (44.1)		306 (35.8)	
Parity								
1	837 (44.4)		1,061 (53.8)		544 (29.0)		387 (25.0)	
2	647 (41.3)	<0.001	806 (48.2)	<0.001	451 (29.1)	<0.001	277 (23.2)	<0.001
3	284 (36.7)		382 (44.4)		173 (21.5)		111 (17.9)	
≥4	147 (28.0)		165 (26.3)		70 (13.2)		55 (14.9)	
Family size								
≤5	966 (40.3)	0.81	1,243 (47.9)	0.13	633 (26.1)	0.72	402 (21.0)	0.182
>5	949 (40.4)		1,171 (46.1)		605 (26.1)		428 (23.5)	
Use of contraception							
Yes	1,312 (40.9)	0.24	1,677 (48.5)	<0.001	832 (26.0)	0.8	566 (22.3)	1.00
No	603 (39.2)		737 (44.1)		406 (26.2)		264 (22.1)	

*p-value from chi-square test; unweighted frequency; weighted percentage.

Table 2 shows the percent distribution of women taking ANC according to their pregnancy intention and other socio-demographic characteristics. Significantly higher proportion of women whose pregnancy was intended received their first ANC visit within the first 3 months of their pregnancy (42.0% vs. 33.9%), received four or more ANC visits (49.2% vs. 38.7%), received all ANC services (27.4% vs. 21.1%), and received adequate IFA supplements (23.3% vs. 17.8%) compared to those whose pregnancy was unintended. Women from urban areas, belonging to the richest wealth quintile, with higher secondary or more education, with partners having higher secondary education or more, and with 1 parity were found to have higher levels of taking the first ANC visit within the 3 months, receiving four or more ANC visits, receiving all ANC services, and taking adequate IFA supplements. A higher percentage of women currently having no employment received their first ANC visit within 3 months and all ANC services, while a higher percentage of women currently using contraception received four or more ANC visits during their last pregnancy. Moreover, a higher level of women from the Dhaka division received their first ANC visit within the first 3 months, all ANC services, and adequate IFA supplements, while the percentage of women receiving four or more ANC visits was higher among women from the Rangpur division.

### Association of Pregnancy Intention and Other Socio-Demographic Variables With ANC

Maternal pregnancy intention and other socio-demographic factors of ANC are presented in Table 3. After controlling for potential confounding socio-demographic factors, it was found that women with unintended pregnancy were 22% less likely to receive their first ANC visit within the first 3 months (AOR: 0.78, 95% CI: 0.66–0.93, *p* = 0.004) than those with intended pregnancy. Similarly, women with unintended pregnancy were less likely to receive four or more ANC visits (AOR: 0.78, 95% CI: 0.66–0.91, *p* = 0.002) and to receive all ANC services (AOR: 0.78, 95% CI: 0.65–0.95, *p* = 0.015) than women with intended pregnancy. Although pregnancy intention was significantly related to taking adequate IFA supplements in bivariate analysis, no significant association was found after adjusting for possible socio-demographic factors in multivariate analysis. All other socio-demographic determinants of ANC were the age of the women, educational level of the women and their partner, type of residential area, division, parity, and wealth quintiles. Receiving the first ANC visit within 3 months, receiving four or more ANC visits, taking all ANC services, and taking adequate IFA supplements tended to be higher with their educational level, their partner’s educational level, and wealth quintiles. In contrast, all these components of ANC tended to be lower with increasing parity. Women aged 25–34 years were more likely to receive their first ANC visit within the first 3 months (AOR: 1.23, 95% CI: 1.04–1.45, *p* = 0.017), to receive four or more ANC visits (AOR: 1.29, 95% CI: 1.10–1.52, *p* = 0.002) to receive all ANC services (AOR: 1.29, 95% CI: 1.08–1.54, *p* = 0.005) and to take adequate IFA supplements (AOR: 1.37, 95% CI: 1.11–1.69, *p* = 0.003) than women aged 15–24 years. In addition, women from urban areas were more likely to receive their first ANC visit within the first 3 months (AOR: 1.17, 95% CI: 1.01–1.35, *p* = 0.042), to receive four or more ANC visits (AOR: 1.34, 95% CI: 1.16–1.54, *p* < 0.001) and to receive all ANC services (AOR: 1.23, 95% CI: 1.05–1.45, *p* = 0.01) than women from rural areas.

**TABLE 3:** Logistic regression of pregnancy intention and other factors associated with antenatal care among women who had a live birth in the 3 years preceding the survey (Bangladesh, 2018).

Variables	First ANC visit within 3 months AOR (95% CI)^a^	*p*-value	Received at least 4 ANC visits AOR (95% CI)^a^	*p*-value	Received all the ANC services AOR (95% CI)^a^	*p*-value	Received adequate IFA AOR (95% CI)^a^	*p*-value
Intention for pregnancy							
Intended	1		1		1		1	
Unintended	0.78 (0.66, 0.93)	0.004	0.78 (0.66, 0.91)	0.002	0.78 (0.65, 0.95)	0.015	1.22 (0.97, 1.52)	0.084
Age of the women (in years)								
15–24	1		1		1		1	
25–34	1.23 (1.04, 1.45)	0.017	1.29 (1.10, 1.52)	0.002	1.29 (1.08, 1.54)	0.005	1.37 (1.11, 1.69)	0.003
≥35	1.18 (0.85, 1.64)	0.32	1.35 (0.98, 1.86)	0.068	1.28 (0.89, 1.85)	0.19	1.67 (1.12, 2.50)	0.012
Area of residence							
Rural	1		1		1		1	
Urban	1.17 (1.01, 1.35)	0.042	1.34 (1.16, 1.54)	<0.001	1.23 (1.05, 1.45)	0.010	1.03 (0.85, 1.25)	0.744
Region (division)								
Barisal	1		1		1		1	
Chattagram	0.78 (0.60, 1.02)	0.06	0.83 (0.65, 1.06)	0.13	0.84 (0.63, 1.11)	0.22	1.67 (1.17, 2.38)	0.005
Dhaka	1.38 (1.06, 1.80)	0.017	1.19 (0.92, 1.53)	0.19	0.98 (0.73, 1.30)	0.86	1.84 (1.28, 2.64)	0.001
Khulna	1.13 (0.86, 1.49)	0.38	1.89 (1.44, 2.47)	<0.001	1.05 (0.78, 1.42)	0.73	1.48 (1.01, 2.16)	0.044
Mymensingh	1.30 (0.99, 1.70)	0.06	1.59 (1.23, 2.06)	<0.001	0.95 (0.70, 1.28)	0.72	2.05 (1.42, 2.96)	<0.001
Rajshahi	0.95 (0.72, 1.26)	0.73	1.38 (1.06, 1.80)	0.016	0.83 (0.61, 1.13)	0.23	1.08 (0.73, 1.61)	0.704
Rangpur	1.17 (0.89, 1.54)	0.25	2.84 (2.18, 3.70)	<0.001	1.04 (0.77, 1.41)	0.8	1.41 (0.97, 2.04)	0.074
Sylhet	1.67 (1.28, 2.18)	<0.001	0.90 (0.70, 1.17)	0.44	0.87 (0.65, 1.18)	0.38	1.22 (0.83, 1.81)	0.311
Educational level of the women							
No education	1		1		1		1	
Primary	0.96 (0.69, 1.34)	0.82	1.69 (1.23, 2.32)	0.001	1.24 (0.81, 1.91)	0.32	0.86 (0.52, 1.44)	0.570
Secondary	1.17 (0.84, 1.62)	0.37	2.38 (1.73, 3.27)	<0.001	1.42 (0.93, 2.17)	0.1	1.70 (1.03, 2.82)	0.038
Higher secondary	1.81 (1.24, 2.63)	0.002	2.86 (1.98, 4.13)	<0.001	2.07 (1.30, 3.28)	0.002	2.27 (1.32, 3.90)	0.003
Employment status of women								
Yes	0.93 (0.80, 1.06)	0.28	—		0.97 (0.83, 1.13)	0.68	0.93 (0.78, 1.12)	0.440
No	1				1		1	
Educational level of the partner								
No education	1		1		1		1	
Primary	1.06 (0.85, 1.33)	0.61	1.10 (0.89, 1.36)	0.37	0.92 (0.69, 1.21)	0.52	0.91 (0.65, 1.26)	0.553
Secondary	1.42 (1.12, 1.80)	0.004	1.41 (1.13, 1.75)	0.003	1.22 (0.92, 1.62)	0.17	1.00 (0.71, 1.41)	0.994
Higher secondary	1.97 (1.49, 2.61)	<0.001	1.96 (1.50, 2.58)	<0.001	1.54 (1.11, 2.12)	0.009	1.31 (0.90, 1.92)	0.163
Wealth quintile								
Poorest	1		1		1		1	
Second	1.29 (1.05, 1.60)	0.017	1.22 (1.01, 1.49)	0.043	1.31 (1.00, 1.71)	0.047	1.18 (0.88, 1.58)	0.283
Middle	1.38 (1.11, 1.71)	0.004	1.63 (1.33, 2.00)	<0.001	1.69 (1.29, 2.20)	<0.001	1.05 (0.77, 1.43)	0.752
Fourth	1.31 (1.05, 1.64)	0.017	1.85 (1.50, 2.29)	<0.001	2.02 (1.55, 2.63)	<0.001	1.13 (0.83, 1.54)	0.437
Richest	2.36 (1.84, 3.04)	<0.001	3.03 (2.37, 3.87)	<0.001	2.84 (2.13, 3.80)	<0.001	1.61 (1.16, 2.26)	0.005
Parity							
1	1		1		1		1	
2	0.94 (0.79, 1.11)	0.44	0.79 (0.67, 0.93)	0.004	1.02 (0.85, 1.22)	0.8	0.86 (0.70, 1.06)	0.163
3	0.84 (0.66, 1.06)	0.13	0.76 (0.61, 0.95)	0.018	0.78 (0.60, 1.01)	0.055	0.69 (0.51, 0.94)	0.018
≥4	0.75 (0.56, 1.01)	0.06	0.51 (0.38, 0.68)	<0.001	0.57 (0.40, 0.81)	0.002	0.65 (0.43, 0.98)	0.039
Family size								
≤5	—		1		—		1	
>5			0.98 (0.86, 1.11)	0.74			1.10 (0.93, 1.30)	0.269
Use of contraception								
Yes	1.07 (0.93, 1.23)	0.34	1.14 (1.0, 1.30)	0.052	—		—	—
No	1		1				

^a^ AOR, adjusted odds ratio; CI, confidence interval.

### Socio-Demographic Factors of ANC Among Women With Intended and Unintended Pregnancy

Women’s age, educational level, partner’s educational level, wealth index, parity, and region had significant association with the utilization of ANC among women with intended pregnancy (Supplementary Table S2). ANC utilization among these women tended to increase with their higher educational level, partner’s higher educational level, and higher wealth quintiles. However, receiving adequate ANC tended to be lower with the increase of parity. On the other hand, among the women with unintended pregnancy, receiving at least 4 ANC visits tended to be higher with the educational level of the women (Supplementary Table S3). Other ANC indicators were not significantly related to their educational level. Their partner’s educational level had no significant relation with ANC utilization among women with unintended pregnancy. However, ANC utilization among these women tended to increase with higher wealth quintiles.

## Discussion

This study highlights the association of maternal pregnancy intention with their utilization of ANC among women aged 15–49 years who had a live birth in the 3 years preceding the 2018 BDHS survey in Bangladesh. In this study, approximately one-fifth of the women reported that their last pregnancy was unintended (wanted later/not wanted at all). Age of the women, residence area, educational level of the women, wealth index, family size, and currently using of contraception significantly influence their pregnancy intention. About 8% of the women did not have any ANC visits during their last pregnancy. Among those who had at least one ANC visit, approximately two-fifth (40.4%) initiated their first visit within the first trimester (first 3 months) of their pregnancy. About half of them had at least four ANC visits, and more than three-fourths of them received their supplementary iron tablets/syrup during their previous pregnancy. However, the adequacy of IFA dosage was only among one-fifth of them (22.2%). Moreover, only one-fourth of them received all ANC services (taking body weight, checking blood pressure, testing blood samples, testing urine samples, performing ultrasonograms, and giving information on pregnancy complications to the mother). The multivariate analyses showed that women with unintended pregnancy were less likely to receive their first ANC visit within the first 3 months, to receive four or more ANC visits and to receive all ANC services than those with intended pregnancy. However, receiving adequate IFA supplements during pregnancy did not significantly vary with pregnancy intention. Other determinants of the ANC were the age of the women, educational level of the women and their partner, type of residential area, division, parity, and wealth quintiles.

Consistent with previous studies [11, 14, 17, 18, 26], our findings showed that women who conceived unintentionally were late in initiating ANC visits to a qualified health professional. This association could be explained in several ways. First, when it is unintended pregnancy, they might be late in recognizing it, and in corollary, they are late in taking their first ANC visit [27]. Another explanation could be that when they conceive unintentionally, they might be mentally unprepared, disturbed, and afraid of sharing it with their partners, family members, or others and confused about whether it should be taken or aborted. Moreover, they might not be financially prepared to take ANC from a qualified health professional. Hence, they are late in initiating the first ANC visit to a qualified health professional during an unwelcome pregnancy.

In addition, our study findings also highlighted that the adequacy of ANC visits (i.e., at least four ANC visits) and the quality of ANC services among pregnant women were also linked with their pregnancy intention. Women with unintended pregnancies were less likely to receive four or more ANC visits, and all ANC services than women with intended pregnancies. Similar findings were reported by previous studies in Bangladesh [11, 14] and other LMICs [17, 28, 29]. One possible reason could be their emotional and financial unpreparedness for meeting the demands of pregnancy, childbearing, and care of themselves and the developing fetus during pregnancy. Moreover, inadequate support and nonchalant attitudes of the partner and other family members toward this unwelcome pregnancy might restrict them from taking adequate maternal care from a qualified health professional. However, maternal pregnancy intention was not significantly linked with taking adequate IFA supplements during pregnancy. This finding was analogous to a study in Ethiopia [30] that concluded that taking supplementary iron tablets during pregnancy did not significantly vary with maternal pregnancy intention. Although the coverage of IFA supplementation was higher, the adequacy of the dosage of IFA was poor. The possible cause behind the high coverage of IFA supplements, irrespective of their pregnancy intention, could be the free and wide availability of iron and folic acid tablets at the local pharmacies, community clinics and public hospitals in Bangladesh. Evidence warns that late initiation of ANC visits, inadequate ANC visits, inadequate ANC services, and inadequate IFA tablets might cause complications during pregnancy, delivery, maternal mortality, still birth, low birth weight (LBW), neonatal mortality, etc. [3, 4, 31]. Therefore, health providers, especially at the community level, could be trained to identify unintended pregnancies and provide culturally appropriate support and care to women with unintended pregnancies.

Socio-demographic factors such as age, type of residential area, educational level of the women, educational level of their partner, wealth index, parity, etc., were significantly linked with the utilization of ANC. Comparatively educated women and women with an educated partner were more likely to receive their first ANC visit within the first 3 months of pregnancy, to receive at least four ANC visits, to receive all the components of ANC services, and to take supplementary iron tablets. Similar findings were found in previous studies in Bangladesh [11, 12, 14, 16, 20, 23, 32, 33] and other LMICs [13, 22, 29, 30, 32, 34]. The educated women and their educated partner might have adequate knowledge about maternal healthcare and are aware of the importance of taking sufficient ANC, which in turn might improve their health-seeking behavior, leading to greater utilization of an optimum level of healthcare during pregnancies [20, 35]. Moreover, educated mothers are more likely to be empowered and have decision-making power within the household regarding their pregnancies and their own healthcare. Therefore, policies aimed at improving the educational level of the mother, their partner, and other family members could be emphasized to foster the utilization of optimum care of mothers during pregnancies.

Consistent with previous studies in Bangladesh [11, 12, 14, 16, 20, 23, 36] and other LMICs [13, 22, 29, 37–41], our findings revealed that household wealth status is a significant predictor of ANC utilization. Receiving the first ANC visit within 3 months, receiving four or more ANC visits, taking all ANC services, and taking adequate supplementary tablets/syrup tended to be higher with their wealth quintiles. This relation was significant irrespective of their pregnancy intention. Higher wealth status had positive effect in ANC utilization among both groups of women (with intended and unintended pregnancy). Measures aimed at improving the living standard of the people, reducing the cost of taking maternal healthcare, and providing incentives could be taken to optimize the utilization of ANC by health professionals.

In agreement with other studies [12–14, 16, 20, 22, 23, 39], our study findings showed that as the number of children of a women increases, the utilization of optimum ANC from health professionals decreases. The cause of this negative association between the women’s parity and utilization of optimum ANC could be their experience from the previous pregnancy, the cost of healthcare in the previous pregnancy, and less excitement during the current pregnancy compared to earlier pregnancy [42].

Moreover, women from rural areas were less likely to receive optimum healthcare services during their pregnancies compared to women from urban areas. Similar findings were also reported in previous studies [13, 16, 20, 22, 23, 38, 41]. The lower utilization of adequate ANC services among rural women might be linked to their lower economic condition, unawareness, superstitions, limited availability of health facilities, etc. Policies to improve health facilities at rural community clinics and raising awareness regarding maternal healthcare could be an appropriate option to facilitate the optimum utilization of ANC services during pregnancy.

The study has strengths as well as some unavoidable limitations. One of its strengths lies in its nationally representative survey data, which makes it possible to generalize the findings at the national and sub-national levels. This study extends the previous work on the effect of maternal pregnancy intention and ANC utilization using all the important ANC indicators. Moreover, the study findings might have important policy implications for further improvement of healthcare services and their optimal utilization during pregnancies. Since unintended pregnancy has a negative effect on the utilization of ANC, policies could be taken to promote the effective use of contraceptives to reduce unintended pregnancy. The study also indicated that the educational of the women could contribute to reduce unintended pregnancies and to foster the uptake of proper ANC. Thus, the findings of the study could be a guide to enhance health education for women especially focusing on the importance and ways of family planning, proper pregnancy care. Nevertheless, the study should be considered with some limitations. Since the study was based on retrospective data on pregnancy intention and ANC seeking, it might impose recall bias. Moreover, the study only reports pregnancy intention and antenatal care seeking behavior of the women with a live birth 3 years preceding the survey. As a result, the information of women who terminated the pregnancy and women who still had a birth was unknown. In addition, due to the cross-sectional nature of the data, the study can only provide evidence of statistical association, and cause-effect relationships cannot be inferred. However, as suggested by Markovitaz et al. [43], cross-sectional studies may be a better source of data for policy judgments in the public health community than longitudinal studies when risk factors vary more across space at a fixed moment in time than at a fixed location across time. Lastly, we could not include other variables that might have explained our outcome variables (i.e.; cost of care, availability, and accessibility of health facilities) because we used secondary data.

### Conclusion

The study revealed that proper antenatal care utilization was inadequate among pregnant women in Bangladesh. Moreover, unintended pregnancy had a negative effect on the adequate utilization of ANC among these women. This inadequate healthcare seeking behavior during the critical period of pregnancy might have a significant adverse effect on maternal and child health, leading to maternal and neonatal death. Appropriate measures to reduce unintended pregnancy might foster the utilization of optimum antenatal care. Policies could be taken to promote the effective use of contraceptives to reduce unintended pregnancy, and health providers, especially at the community level, could be trained on how to identify unintended pregnancies and provide culturally appropriate support and care to women with unintended pregnancies. Moreover, to foster the utilization of optimum care of women during pregnancies, appropriate measures aimed at improving the educational level of the women, their partner, and other family members and improving the living standards of the people, reducing the cost of taking maternal healthcare, and providing incentives could be emphasized. Health education for women especially focusing on the importance of family planning, and proper pregnancy care and how to adopt them, could be another approach to reduce unintended pregnancy and increase adequate antenatal care.

## Data Availability

The relevant data of this was obtained from MEASURE DHS and are available from the Demographic Health Surveys Program (Bangladesh Standard DHS, 2017- https://dhsprogram.com/data/dataset/Bangladesh_Standard-DHS_2017.cfm?flag=1).
