# Supplementary material for: Maternal Pregnancy Intention and Antenatal Care Seeking Behaviors in Bangladesh: Evidence From Bangladesh Demographic and Health Survey, 2018
Source: Int J Public Health. 2023 Jul 11;68:1605944. doi: 10.3389/ijph.2023.1605944 (PMC10366356; doi:10.3389/ijph.2023.1605944)
Supplement: Supplementary file 1 [file DataSheet1.docx]

Supplementary Table 1: Pregnancy intention and socio-demographic characteristics of the women aged 15-49 years who had a live birth in the three years preceding the survey (n = 5012) (Bangladesh, 2018)

| **Variables** | **Pregnancy intention** | | **Unintended pregnancy** | |
| --- | --- | --- | --- | --- |
|  | **Intended** | **Unintended** | **AOR (95% CI)** | **p value** |
| Age of the women (in years)  15-24  25-34  ≥35 | 2173 (82.3)  1593 (77.7)  188 (60.2) | 469 (17.7)  466 (22.3)  123 (39.8) | 1  1.39 (1.19, 1.61)  2.81 (2.15, 3.77) | <0.001  <0.001 |
| Area of residence  Rural  Urban | 2604 (79.6)  1350 (77.8) | 683 (20.4)  375 (22.2) | 1  0.81 (0.68, 0.95) | 0.01 |
| Region (division)  Barisal  Chattagram  Dhaka  Khulna  Mymensingh  Rajshahi  Rangpur  Sylhet | 404 (75.3)  697 (83.6)  587 (79.1)  389 (74.1)  485 (80.4)  421 (80.1)  422 (74.6)  549 (79.0) | 129 (24.7)  138 (16.4)  154 (20.9)  135 (25.9)  118 (19.6)  106 (19.9)  137 (25.4)  141 (21.0) | 1  0.67 (0.51, 0.89)  0.90 (0.68, 1.20)  1.15 (0.86, 1.53)  0.73 (0.55, 0.98)  0.83 (0.62, 1.13)  1.02 (0.76, 1.35)  0.76 (0.58, 1.01) | 0.005  0.48  0.36  0.038  0.24  0.91  0.063 |
| Educational level of the women  No education  Primary  Secondary  Higher secondary | 223 (70.9)  1050 (74.3)  1931 (81.7)  750 (82.6 | 89 (29.1)  342 (25.7)  471 (18.3)  156 (17.4) | 1  0.81 (0.61, 1.08)  0.64 (0.48, 0.86)  0.57 (0.39, 0.82) | 0.15  0.003  0.003 |
| Employment status of women  Yes  No | 1433 (76.2)  2521 (80.9) | 447 (23.8)  611 (19.1) | 1.05 (0.90, 1.22)  1 | 0.55 |
| Educational level of the partner  No education  Primary  Secondary  Higher secondary | 503 (73.4)  1285 (77.2)  1308 (81.0)  793 (83.1) | 176 (25.6)  372 (22.8)  327 (19.0)  169 (16.9) | 1  0.99 (0.79, 1.24)  0.99 (0.77, 1.26)  0.92 (0.67, 1.25) | 0.92  0.91  0.58 |
| Wealth quintile  Poorest  Second  Middle  Fourth  Richest | 823 (76.2)  770 (75.2)  731 (81.8)  777 (79.7)  853 (83.1) | 256 (23.8)  247 (24.8)  174 (18.2)  211 (20.3)  170 (16.9) | 1  1.07 (0.87, 1.32)  0.80 (0.64, 1.02)  0.90 (0.81, 1.14)  0.62 (0.47, 0.83) | 0.53  0.069  0.35  0.001 |
| Family size  ≤5  >5 | 2051 (81.4)  1903 (76.7) | 473 (18.6)  585 (23.3) | 1  1.46 (1.27, 1.69) | <0.001 |
| Currently using contraception  Yes  No | 2574 (76.6)  1380 (84.0) | 786 (23.4)  272 (16.0) | 1.52 (1.30, 1.79)  1 | <0.001 |

*AOR: Adjusted odds ratio; CI: Confidence interval; unweighted frequency; weighted percentage; p value from multivariate logistic regression

Supplementary Table 2: Socio-demographic determinants of antenatal care among the women whose pregnancy was intended (Bangladesh, 2018)

| **Variables** | **First ANC visit within three months** | | **Received at least 4 ANC visits** | | **Received all the ANC services** | | **Adequate IFA consumption** | |
| --- | --- | --- | --- | --- | --- | --- | --- | --- |
|  | **n (%)** | **AOR (95% CI)** | **n (%)** | **AOR (95% CI)** | **n (%)** | **AOR (95% CI)** | **n (%)** | **AOR (95% CI)** |
| Total | 1587 (42.0) |  | 1985 (49.2) |  | 1036 (27.4) |  | 697 (23.3) |  |
| Age of the women (in years)  15-24  25-34  ≥35 | 843 (41.3)  667 (43.2)  77 (38.9) | 1  1.18 (0.99, 1.43)  1.23 (0.83, 1.84) | 1072 (48.5)  823 (50.8)  90 (42.9) | 1  1.27 (1.06, 1.52)*  1.25 (0.85, 1.85) | 535 (26.1)  455 (29.2)  46 (25.9) | 1  1.27 (1.05, 1.55)*  1.16 (0.75, 1.79) | 361 (22.0)  296 (24.9)  40 (26.2) | 1  1.30 (1.03, 1.63)*  1.60 (1.01, 2.55)* |
| Area of residence  Rural  Urban | 899 (37.7)  688 (53.6) | 1  1.25 (1.06, 1.47)** | 1153 (61.7)  832 (44.7) | 1  0.73 (0.62, 0.86)*** | 549 (23.4)  487 (37.9) | 1  1.28 (1.06, 1.52)** | 403 (21.1)  294 (28.8) | 1  1.06 (0.86, 1.31) |
| Region (division)  Barisal  Chattagram  Dhaka  Khulna  Mymensingh  Rajshahi  Rangpur  Sylhet | 134 (36.5)  237 (35.9)  300 (52.2)  173 (42.9)  180 (39.4)  149 (34.2)  174 (38.4)  240 (47.9) | 1  0.80 (0.60, 1.07)  1.43 (1.06, 1.92)*  1.24 (0.90, 1.70)  1.22 (0.90, 1.66)  0.96 (0.70, 1.32)  1.25 (0.91, 1.71)  1.71 (1.26, 2.31)** | 155 (36.0)  300 (41.1)  331 (54.3)  243 (59.7)  246 (47.7)  218 (49.0)  278 (63.7)  214 (36.4) | 1  0.99 (0.75, 1.31)  1.40 (1.05, 1.86)*  2.32 (1.70, 3.17)***  1.93 (1.44, 2.58)***  1.65 (1.22, 2.22)**  3.61 (2.65, 4.91)***  1.04 (0.78, 1.39) | 88 (24.2)  180 (26.4)  195 (33.2)  125 (31.4)  113 (23.1)  99 (22.8)  113 (24.6)  123 (22.9) | 1  0.95 (0.69, 1.30)  1.09 (0.79, 1.50)  1.22 (0.87, 1.72)  1.13 (0.80, 1.59)  0.92 (0.65, 1.30)  1.16 (0.82, 1.64)  0.99 (0.70, 1.39) | 41 (16.1)  133 (25.0)  136 (27.9)  76 (24.1)  100 (26.4)  56 (14.7)  84 (22.1)  71 (17.4) | 1  1.71 (1.14, 2.56)*  1.95 (1.30, 2.94)**  1.63 (1.05, 2.52)*  2.28 (1.50, 3.45)**  1.07 (0.68, 1.69)  1.57 (1.03, 2.40)*  1.38 (0.89, 2.14) |
| Educational level of the women  No formal education  Primary  Secondary  Higher secondary | 56 (32.3)  283 (30.1)  762 (40.6)  486 (63.5) | 1  0.78 (0.54, 1.23)  0.99 (0.68, 1.43)  1.57 (1.03, 2.39)* | 52 (22.8)  377 (36.2)  1020 (51.5)  536 (69.9) | 1  1.55 (1.09, 2.21)*  2.16 (1.51, 3.10)***  2.54 (1.69, 2.83)*** | 119 (11.9)  174 (18.2)  492 (26.4)  351 (45.5) | 1  1.63 (0.97, 2.74)  1.74 (1.04, 2.92)*  2.48 (1.43, 4.31)** | 18 (13.1)  87 (13.1)  343 (22.8)  249 (38.1) | 1  0.84 (0.47, 1.47)  1.57 (0.90, 2.74)  2.05 (1.13, 3.73)* |
| Employment status of women  Yes  No | 494 (36.3)  1093 (45.1) | 0.91 (0.78, 1.07)  1 | 715 (48.2)  1270 (49.7) | - | 325 (23.5)  711 (29.5) | 1.00 (0.84, 1.19)  1 | 228 (20.0)  469 (25.2) | 1.00 (0.82, 1.22) |
| Educational level of the partner  No formal education  Primary  Secondary  Higher secondary | 120 (28.0)  376 (32.2)  557 (42.9)  508 (36.2) | 1  1.09 (0.84, 1.41)  1.45 (1.11, 1.90)**  2.04 (1.49, 2.80)*** | 164 (32.9)  518 (39.0)  690 (51.5)  584 (73.6) | 1  1.10 (0.87, 1.40)  1.36 (1.06, 1.74)*  2.05 (1.52, 2.70)*** | 68 (16.6)  217 (18.1)  378 (29.6)  368 (45.2) | 1  0.93 (0.68, 1.28)  1.31 (0.95, 1.80)  1.67 (1.17, 2.39)** | 52 (16.0)  147 (16.6)  238 (23.3)  253 (36.2) | 1  0.89 (0.62, 1.28)  1.04 (0.71, 1.51)  1.32 (0.87, 2.00) |
| Wealth quintile  Poorest  Second  Middle  Fourth  Richest | 185 (27.2)  252 (35.1)  267 (38.2)  326 (41.4)  557 (64.0) | 1  1.35 (1.06, 1.71)*  1.35 (1.05, 1.72)*  1.35 (1.05, 1.74)*  2.44 (1.84, 3.24)*** | 268 (33.1)  313 (38.6)  355 (46.8)  429 (53.0)  620 (73.4) | 1  1.28 (1.03, 1.60)*  1.58 (1.26, 1.99)***  1.87 (1.40, 2.37)***  3.13 (2.38, 4.11)*** | 89 (12.9)  129 (17.4)  169 (24.7)  249 (32.7)  400 (45.0) | 1  1.35 (1.00, 1.82)  1.72 (1.28, 2.32)***  2.27 (1.69, 3.06)***  3.00 (2.17, 4.14)*** | 80 (16.8)  108 (19.3)  104 (18.4)  137 (21.8)  268 (36.9) | 1  1.20 (0.86, 1.66)  1.02 (0.72, 1.44)  1.14 (0.81, 1.60)  1.75 (1.21, 2.52)** |
| Parity  1  2  3  ≥4 | 769 (45.2)  520 (42.2)  212 (39.1)  86 (27.7) | 1  0.93 (0.77, 1.11)  0.89 (0.69, 1.16)  0.74 (0.52, 1.06) | 960 (54.0)  646 (49.2)  273 (45.2)  106 (31.2) | 1  0.79 (0.66, 0.94)**  0.77 (0.60, 0.99)*  0.61 (0.44, 0.86)** | 500 (29.6)  370 (29.7)  121 (21.1)  45 (15.8) | 1   - 1. (0.83, 1.24)   0.73 (0.54, 0.97)*  0.63 (0.41, 0.95)* | 350 (25.1)  236 (25.0)  74 (16.8)  37 (17.4) | 1  0.93 (0.74, 1.16)  0.65 (0.46, 0.91)*  0.77 (0.48, 1.23) |
| Family size  ≤5  >5 | 816 (41.6)  771 (42.4) | - | 1036 (49.4)  949 (49.0) | - | 532 (26.8)  504 (28.0) | - | 347 (22.3)  350 (24.5) | - |

AOR: Adjusted odds ratio from multivariate logistic regression; CI: Confidence interval;

*Significant at p-value < 0.05; **Significant at p-value < 0.01; Significant at p-value < 0.001

Supplementary Table 3: Socio-demographic determinants of antenatal care among the women whose pregnancy was unintended (Bangladesh, 2018)

| **Variables** | **First ANC visit within three months** | | **Received at least 4 ANC visits** | | **Received all the ANC services** | | **Adequate IFA consumption** | |
| --- | --- | --- | --- | --- | --- | --- | --- | --- |
|  | **n (%)** | **AOR (95% CI)** | **n (%)** | **AOR (95% CI)** | **n (%)** | **AOR (95% CI)** | **n (%)** | **AOR (95% CI)** |
| Total | 328 (33.9) |  | 429 (38.7) |  | 202 (21.1) |  | 133 (17.8) |  |
| Age of the women (in years)  15-24  25-34  ≥35 | 146 (32.8)  152 (36.4)  30 (28.7) | 1  1.42 (0.96, 2.09)  1.20 (0.65, 2.22) | 202 (40.7)  187 (38.3)  40 (32.3) | 1  1.41 (0.97, 2.04)  1.72 (0.94, 3.12) | 90 (20.7)  92 (21.9)  20 (19.2) | - | 57 (17.0)  63 (18.5)  13 (18.1) | - |
| Area of residence  Rural  Urban | 200 (32.9)  128 (36.3) | 1  1.09 (0.78, 1.52) | 243 (34.9)  186 (48.4) | 1  1.27 (0.92, 1.72) | 112 (18.3)  90 (27.6) | 1  0.94 (0.65, 1.38) | 78 (15.6)  55 (23.2) | 1  1.02 (0.65, 1.62) |
| Region (division)  Barisal  Chattagram  Dhaka  Khulna  Mymensingh  Rajshahi  Rangpur  Sylhet | 38 (33.8)  31 (27.4)  53 (39.3)  39 (29.6)  46 (43.4)  36 (32.7)  37 (28.2)  48 (38.6) | 1  0.69 (0.38, 1.26)  1.20 (0.67, 2.14)  0.86 (0.48, 1.54)  1.69 (0.94, 3.04)  0.95 (0.52, 1.74)  0.91 (0.51, 1.64)  1.49 (0.83, 2.68) | 59 (43.7)  38 (25.8)  64 (39.6)  69 (49.1)  45 (35.8)  48 (43.4)  66 (46.1)  40 (27.5) | 1  0.43 (0.24, 0.76)**  0.72 (0.41, 1.26)  1.03 (0.60, 1.76)  0.85 (0.48, 1.48)  0.84 (0.47, 1.49)  1.42 (0.84, 2.43)  0.57 (0.32, 1.01) | 32 (30.6)  22 (18.0)  33 (23.5)  29 (20.0)  17 (17.2)  22 (21.9)  26 (20.5)  21 (16.9) | 1  0.54 (0.28, 1.05)  0.63 (0.33, 1.21)  0.63 (0.34, 1.19)  0.45 (0.23, 0.91)*  0.56 (0.29, 1.09)  69 (0.37, 1.31)  0.54 (0.27, 1.08) | 15 (16.5)  24 (25.1)  22 (19.3)  17 (16.6)  16 (19.5)  14 (16.6)  15 (11.8)  10 (11.9) | 1  2.05 (0.94, 4.43)  1.77 (0.79, 3.98)  1.13 (0.50, 2.52)  1.55 (0.68, 3.54)  1.34 (0.57, 3.13)  1.13 (0.49, 2.59)  0.92 (0.36, 2.35) |
| Educational level of the women  No formal education  Primary  Secondary  Higher secondary | 11 (13.5)  88 (30.5)  149 (33.3)  80 (52.1) | 1  1.85 (0.89, 3.86)  1.89 (0.88, 4.02)  2.37 (0.98, 5.75) | 11 (11.9)  99 928.0)  216 (44.0)  103 (64.8) | 1  2.31 (1.13, 4.75)*  3.33 (1.60, 6.93)**  4.60 (1.93, 10.93)** | 11 (16.8)  38 (12.9)  93 (20.8)  60 (40.9) | 1  0.60 (0.27, 1.32)  0.84 (0.38, 1.86)  1.39 (0.55, 3.51) | 4 (12.3)  18 (7.5)  66 (20.0)  45 (33.1) | 1  0.88 (0.27, 2.88)  0.21 (0.66, 6.74)  3.11 (0.85, 11.36) |
| Employment status of women  Yes  No | 126 (30.7)  202 (36.3) | 1.02 (0.75, 1.39) | 173 (36.6)  256 (40.3) | - | 71 (17.9)  131 (23.4) | 0.88 (0.61, 1.27) | 43 (1.38)  90 (20.9) | 0.68 (0.44, 1.05) |
| Educational level of the partner  No formal education  Primary  Secondary  Higher secondary | 36 (23.8)  88 (27.3)  112 (35.9)  91 (55.3) | 1  0.97 (0.60, 1.57)  1.25 (0.75, 2.08)  1.76 (0.91, 3.39) | 39 (21.1)  119 (31.0)  159 (46.9)  110 (63.8) | 1  1.13 (0.71, 1.78)  1.57 (0.97, 2.55)  1.49 (0.78, 2.83) | 21 (17.1)  50 (15.0)  67 (20.7)  63 (39.8) | 1  0.88 (0.49, 1.60)  0.97 (0.52, 1.81)  1.16 (0.54, 2.49) | 10 (9.2)  34 (14.1)  41 (17.3)  48 (33.0) | 1  1.12 (0.50, 2.48)  1.02 (0.44, 2.34)  1.50 (0.57, 3.97) |
| Wealth quintile  Poorest  Second  Middle  Fourth  Richest | 52 (25.8)  60 (28.2)  61 (34.1)  69 (35.1)  86 (50.2) | 1  1.18 (0.75, 1.85)  1.56 (0.96, 2.54)  1.25 (0.76, 2.04)  2.16 (1.21, 3.87)** | 65 (23.7)  74 (29.6)  78 (39.9)  103 (47.1)  109 (63.4) | 1  1.05 (0.69, 1.60)  1.71 (1.08, 2.73)*  1.87 (1.17, 2.98)**  2.72 (1.55, 4.97)** | 27 (13.1)  33 (13.9)  38 (21.3)  40 (21.9)  64 (39.4) | 1  1.21 (0.69, 2.14)  1.68 (0.93, 3.03)  1.33 (0.73, 2.44)  2.65 (1.35, 5.19)** | 17 (10.0)  22 (12.7)  24 (17.5)  32 (21.8)  38 (30.0) | 1  1.15 (0.57, 2.31)  1.34 (0.65, 2.79)  1.23 (0.59, 2.55)  1.35 (0.59, 3.09) |
| Parity  1  2  3  ≥4 | 68 (37.6)  127 (37.9)  72 (30.6)  61 (28.5) | 1  0.95 (0.62, 1.45)  0.67 (0.39, 1.14)  0.65 (0.35, 1.22) | 101 (51.7)  160 (44.3)  109 (42.4)  59 (20.7) | 1  0.79 (0.53, 1.20)  0.70 (0.42, 1.18)  0.35 (0.19, 0.64) | 44 (23.5)  81 (26.7)  52 (22.5)  25 (10.2) | 1  1.32 (0.83, 2.01)  1.27 (0.76, 2.12)  0.72 (0.38, 1.36) | 37 (24.6)  41 (16.0)  37 (20.4)  18 (11.8) | 1  0.67 (0.39, 1.16)  1.06 (0.60, 1.90)  0.66 (0.32, 1.37) |
| Family size  ≤5  >5 | 150 (34.5)  178 (33.4) | - | 207 (41.3)  222 (36.6) | 0.97 (0.72, 1.30) | 101 (22.9)  101 (19.5) | 0.84 (0.60, 1.18) | 55 (15.5)  78 (19.9) | 1.29 (0.85, 1.96) |

AOR: Adjusted odds ratio from multivariate logistic regression; CI: Confidence interval;

*Significant at p-value < 0.05; **Significant at p-value < 0.01; Significant at p-value < 0.001
